# Exploring Structural and Biological Determinants of Cognitive Health and Aging: Insights From Diverse Populations

**DOI:** 10.1093/geroni/igaf122.2091

**Published:** 2025-12-31

**Authors:** Ruotong Mona Liu, Xiang Qi

## Abstract

Cognitive health and aging are influenced by a complex interplay of social, environmental, and biological factors, yet disparities in these determinants remain underexplored across diverse populations. This symposium brings together four studies that examine how secondhand smoke exposure, central adiposity, racial disparities in autism spectrum disorder (ASD) diagnosis and care, and neighborhood environments shape cognitive and physical health outcomes in aging populations. The first study investigates the impact of secondhand smoke exposure on cognitive function among never-smoking older adults in the U.S., using biomarker-based assessments to highlight the cognitive risks associated with even moderate exposure. The second study explores how body fat distribution modifies the association between obstructive sleep apnea and frailty, revealing significant racial disparities in vulnerability and underscoring the role of central adiposity in aging-related health risks. The third study conducts a scoping review on racial and ethnic disparities in ASD diagnosis and healthcare access among older adults, demonstrating inequities in mental health comorbidities, treatment access, and healthcare utilization. The final study examines the role of cognitively supportive neighborhood environments in slowing cognitive decline among older Chinese Americans over an 8-year period, emphasizing the protective effects of social and environmental resources. Together, these studies contribute to a broader understanding of the structural and biological determinants of cognitive health and aging, offering critical insights for public health policies, community-based interventions, and personalized healthcare strategies aimed at promoting health equity and well-being across diverse aging populations.

